# Silicon Oxynitride Thin Film Coating to Lossy Mode Resonance Fiber-Optic Refractometer

**DOI:** 10.3390/s22103665

**Published:** 2022-05-11

**Authors:** Dmitriy P. Sudas, Leonid Yu. Zakharov, Viktor A. Jitov, Konstantin M. Golant

**Affiliations:** 1 World-Class Research Center, Peter the Great St. Petersburg Polytechnical University, Polytechnicheskaya ul. 29, 195251 St. Petersburg, Russia; 2 Kotel’nikov Institute of Radioengineering and Electronics, Russian Academy of Sciences, Fryazino Branch, Sq. Vvedenskogo 1, Fryazino, 141190 Moscow, Russia; l.yu.zakharov@gmail.com (L.Y.Z.); vaz215@googlemail.com (V.A.J.); golant@cplire.ru (K.M.G.)

**Keywords:** lossy mode resonance, fiber sensor, fiber refractometer, SPCVD, fiber taper, silicon oxynitride

## Abstract

A fiber-optic refractometer for various liquids with refractive indices in the range from 1.33 to 1.43 has been manufactured and tested. The sensor is based on a thin silicon oxynitride (Si_3_N_4-x_O_x_) film coated thinned optic fiber section (taper) obtained in a multimode all-silica optical fiber by chemical etching of the reflective cladding. The film was deposited on the cylindrical surface of the thinned fiber by the surface plasma chemical vapor deposition method (SPCVD). Lossy mode resonance (LMR) was observed in the transmission spectrum of the coated taper at a wavelength dependent on the refractive index of the liquid in which the taper was immersed. We tested the obtained sensors in distilled water, isopropyl alcohol, dimethylformamide, and their aqueous solutions. It was found that with the help of the SPCVD, one can obtain a set of sensors in a single deposition run with the dispersion of sensitivity and spectral position of LMR no more than 5%. Maximum sensitivity of the manufactured sensors to surrounding media refractive index (SMRI) variation exceeds 1090 nm/RIU, which is the highest value recorded to date for a sensor with a non-oxide coating.

## 1. Introduction

The refractive index profile of modern optical fibers is designed in such a way that the main portion of light energy transported by the fiber is located in the light-guiding core. However, a small fraction of the energy carrying by the fiber is present in the immediate vicinity of the core-cladding interface. This part of the energy propagating along the core-cladding interface forms the evanescent field of the mode in the cladding region [1]. The evanescent field, due to the coupling effect, can interact with a wide range of coating materials, which affect the propagation of light through the fiber. Based on this phenomenon, devices are being created for application in the fields of fiber lasers [2] and sensors [3,4]. To observe and exploit the effect of the evanescent field interaction with the ambience, geometric change (thinning via etching out, and/or side-polishing) of a local fiber segment are often used [5,6,7]. If the thin-film coating material deposited on such an area were to satisfy certain conditions, then a dip would be observed in the transmission spectrum of the fiber line. For this to take place, the real part of the dielectric permittivity of the coating material must exceed the imaginary part in absolute value. At the same time, it should be greater than corresponding values for the dielectric waveguide and the environment [8]. If the real part of the dielectric permittivity of the coating film is negative, surface plasmon resonance (SPR) will be observed [9] that is typical for metals such as gold [10]. If the real part is positive, then the effect of lossy mode resonance (LMR) will be observed [11]. The latter conditions are satisfied by the widest range of semiconductors [12,13,14,15] and polymers [16] that can be applied to a fiber as thin films. 

The resonant wavelength of the LMR depends on the coating thickness and the refractive index of the surrounding media. The latter allows one to apply the LMR effect to monitor environment refractive index [6,15], pH [17], temperature [18], composition, and even to observe biological objects functioning [16,19].

An important requirement for thin-film coatings intended for fiber sensors primarily becomes strength and resistance to those media with which the sensitive section of the sensor comes into contact. Nitrides are the most stable and durable compounds [20]. Silicon nitride Si_3_N_4_ meets the LMR requirements and already has taken part in sensorics [21]. Due to its antibacterial properties [22] and excellent biogenesis [23,24,25], silicon nitride since recently is actively used in medicine. However, when using this material, surface cracking often takes place [26], due to high internal stresses arising in the synthesis process. In addition, silicon nitride is oxidized in air, and upon contact with organic cells that metabolically assimilate and transform water, carbon dioxide (CO_2_) and three inorganic nitrogen compounds—ammonium (NH^+4^), nitrate (NO^−3^) and nitrogen (N_2_)—into complex biomolecules [27,28,29]. The latter leads to changes in the sensory properties of the final device. One can eliminate this inconvenience by doping or oxidation. Oxynitride films, for example, Si_2_N_2_O, are more stable and resistive to cracking at a low film tension [26]. They are effective diffuse barriers resistant to phosphoric and hydrofluoric acids [26]. 

There are several methods for the synthesis of nitride and oxynitride films. CVD methods require high temperatures in the reaction zone (more than 1000 °C) [30], and, therefore, are of little use for the deposition layers on optical fibers, for which such temperatures are either excessive or close to critical, depending on doping additives. Conventional plasma-chemical methods, such as the technology of plasma enhanced chemical vapor deposition (PECVD [21,31,32]), operating at rather moderate temperatures (about 500 °C) and reduced pressure are preferable. However, these also have a limitation due to the impossibility of uniformly coating the cylindrical surface of the fiber without rotating. This is not a problem for D-shaped fibers [6], in which one side of the lateral surface is removed to the core. However, for LMR-based sensors, it is preferable to use a double adiabatic taper, that is, a fiber section uniformly etched out over a length of several millimeters. In this case, the distance between the coating film and the core remains unchanged over a larger surface area of the fiber taper. This positively affects the shape of the resonance curve and the sensitivity of the sensor. Therefore, a complex rotation system is necessary for uniform coating [32]. However, insufficient homogeneity of the coating film negatively affects the sensor sensitivity in this case as well [32]. In addition, rotation of the fiber carries the risk of breakage. Fortunately, there is a method of applying nitride and oxynitride coatings free of the disadvantages mentioned above.

In this study, we discuss the application of surface plasma chemical vapor deposition (SPCVD) technology [33,34] to the deposition of silicon oxynitride layers on the surface of a chemically thinned fiber section. We demonstrate a promising method for creating sensors based on a unique material using common synthesis technology. 

Section 2 describes the experimental methods that we used to fabricate and characterize fiber sensors. Section 3.1 provides information on the regimes and technological features of sensor fabrication. Data on the surface quality, chemical composition, absorption edge, and optical constants of the synthesized films are presented in Section 3.2. The results of obtained sensors testing in various environments and the possibility of the simultaneous fabrication of several sensors in a single deposition run are discussed in Section 3.3.

## 2. Materials and Methods

For the manufacture of sensors, we used a multimode, all-silica fiber of our own production with a light-guide core with a diameter of 200 μm and a fluorine-doped reflective cladding made of silica with an outer diameter of 220 μm. The reflective cladding was etched out with a low-toxic polishing etchant [35] based on an aqueous solution of ammonium fluoride (NH_4_F) and an ammonium sulfate complexant ((NH_4_)_2_SO_4_) (Merck KGaA, Darmstadt, Germany). For this purpose, the fiber was mounted on a special fluoropolymer plate in a taut state. A drop of etchant was applied to a section of the fiber side surface mechanically released from the protective polymer. The length of the free of protective polymer fiber section did not exceed 5 mm. The composition of the etchant provided a fiber diameter decrease at a rate of 0.5 microns per minute at room temperature. At the end of the process, the etchant was rinsed off by repeatedly washing the fiber surface with distilled water. The surface quality and the thinned section (taper) diameter of the fiber were monitored via an optical microscope. We stopped etching as soon as the diameter of the thinned section of the fiber reached 202–205 microns.

**Figure 1:**
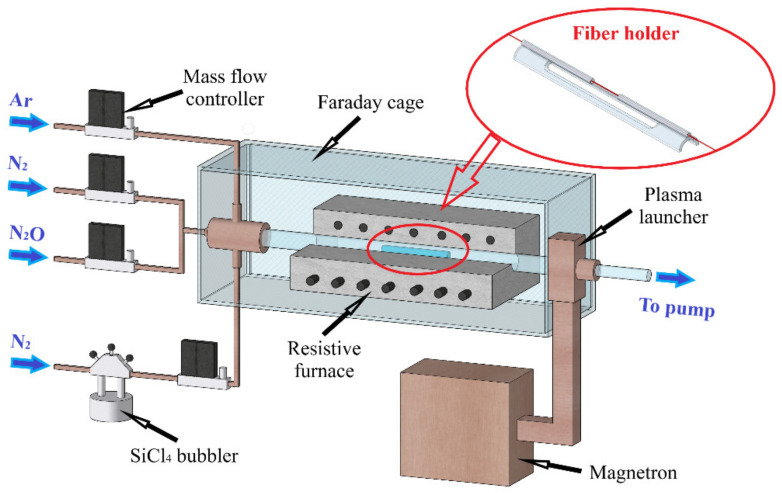
Schematic of the experimental setup for coating film deposition; inset—a model of the fiber holder.

Next, we mounted a piece of fiber with the thinned cladding section in a special holder, as shown in the inset in Figure 1. The fiber stretched in the holder is marked in red.

The holder is designed in such a way that a piece of fiber freely lies in it without fixation being secured by its own weight. After that, the holder with the fiber was placed in a horizontally positioned silica tube-reactor together with a silica plate inside, which was positioned under the fiber at the bottom of the reactor (Figure 1). The distance between the fiber and the plate was set so that no “shadow” effect appeared during the deposition. Before the deposition, the reactor tube with the installed fiber was placed in a horizontal resistive furnace and evacuated to a pressure of less than 1 Torr. Upon reaching the required pressure, argon and a small amount of nitrogen were fed into the continuously pumped out reactor tube. Then, a stationary microwave discharge in the form of a surface-plasma column was excited in the tube, and the system for its length periodic changing was activated via variation of the microwave power supplied to the plasma. A periodic change in the supplied microwave power with a frequency of 4 Hz scanned the end of the plasma column in such a way as to ensure the passage of the deposition zone through the place in the substrate tube with the holder containing the fiber taper mounted in it.

The composition of the plasma was monitored by the emission spectrum in the visible wavelength range using a Compact CCD Spectrometer CCS100 (Thorlabs Inc., Newton, NJ, USA). The temperature of the tube surface was monitored with an IRCON SR (Fluke Co., Beaverton, OR, USA). optical pyrometer at the location of the holder with the fiber. When a temperature of 650 °C was reached, vapors of silicon tetrachloride from a bubbler using dried nitrogen as a carrier gas were fed into the tube from the side opposite to the microwave source through the gas flow regulator. The flow rate of the supplied chloride was determined from several comparative experiments with nitrogen bubbling and evaporation only. The temperature of the tank with silicon tetrachloride was kept at the level of 24 °C with fluctuations of less than 1 °C. The source of oxygen was high purity N_2_O (Horst Tec., Zelenograd, Russia) supplied to the reactor. The duration of deposition was set according to the growth rate of the coating, which amounted to 30 nm/min, since the conventional methods of in situ thickness control failed due to the high luminosity of the plasma.

To determine the thickness of the coating and study the optical characteristics of the grown film, we first examined the film deposited on a silica glass plate. The transmittance of Si_3_N_4-x_O_x_ coated wafers was measured on a SPECORD UV VIS (Analytik Jena AG, Jena, Germany) spectrometer in the 200–800 nm wavelengths range. The thickness, real and imaginary parts of the dielectric constant of the coating were determined on a homemade ellipsometer. The coating surfaces were tested using a JSM-6480LV (Jeol Ltd., Tokyo, Japan) scanning electron microscope (SEM) with a tungsten thermionic cathode. Raman spectroscopy was performed at room temperature using an XPloRA micro-Raman spectrometer (Horiba Ltd., Kyoto, Japan) excited by a solid-state green laser (λ = 532 nm, power = 10 mW, spot size = 1 μm) in a backscattering configuration.

The sensors removed from the reactor were inserted into a fiber optical transmission measurement circuit. An LS-1 (OceanOptics Inc., Rochester, NY, USA) halogen lamp was used as a light source, and a transmission spectrum in the wavelength range from 900 to 1700 nm was obtained using an NIRQuest-512 spectrometer (OceanOptics Inc., Rochester, NY, USA). The fiber part of the measuring circuit was placed in a fume hood. The film-covered taper was filled into distilled water or a test solution and covered with a fluoroplastic cap to reduce the rate of evaporation of the volatile component of the solution. The measurements were carried out using a set of prepared solutions of isopropyl alcohol and dimethylformammide with a consistently increasing concentration. Before testing the next liquid, the previous mixture was carefully removed from the fiber sensor. The completeness of the purification was assessed by the transmission spectrum.

## 3. Results and Discussion

### 3.1. Coating Application

**Figure 2:**
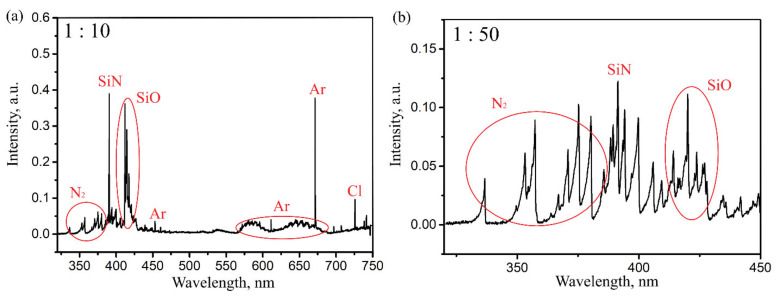
Plasma emission spectra during the synthesis of the oxynitride coating at the ratio of SiCl_4_ to N_2_ in the deposition zone: (**a**) 1:10, (**b**) 1:50.

Figure 2 shows the plasma emission spectra recorded during the synthesis of silicon oxynitride on the surface of the taper. In the first case, the flow rates ratio between SiCl_4_ and N_2_ was 1:10. The N_2_O flow was always set to ensure equality oxygen and silicon concentrations in the plasma. Such ratios between silicon tetrachloride and nitrogen consumptions are insufficient for the formation of a stoichiometric silicon nitride or oxynitride film [36], but are quite suitable to obtain nitrogen-doped silica, for which the SPCVD method has been used for the first time [37]. Figure 2a clearly presents emission lines characteristic of Si-N (~390 nm) and Si-O (~412–420 nm) species. With the fivefold increase in the nitrogen consumption to the reactor necessary for the film deposition, the lines mentioned earlier are no longer so dominant against the nitrogen background, although they are still traced (Figure 2b). Since an increase in the nitrogen content in the plasma leads to more intense heating of the inner surface of the substrate tube and the fiber by plasma, an upper limit of the nitrogen consumption does exist, after which the temperature rises above 700 °C. At such a temperature, the deformation of the fiber starts to occur. For this reason, the necessary ratio between silicon and nitrogen was controlled over reducing the consumption of silicon tetrachloride. After the completion of the deposition process, microwave power was turned off, the reservoir with chloride was shut off, and the entire system continued to be purged with nitrogen and argon until the substrate tube cooled down. Prior to characterization, the synthesized samples were kept in air for a day to stabilize the surface.

### 3.2. Characterization of the Coating

**Figure 3:**
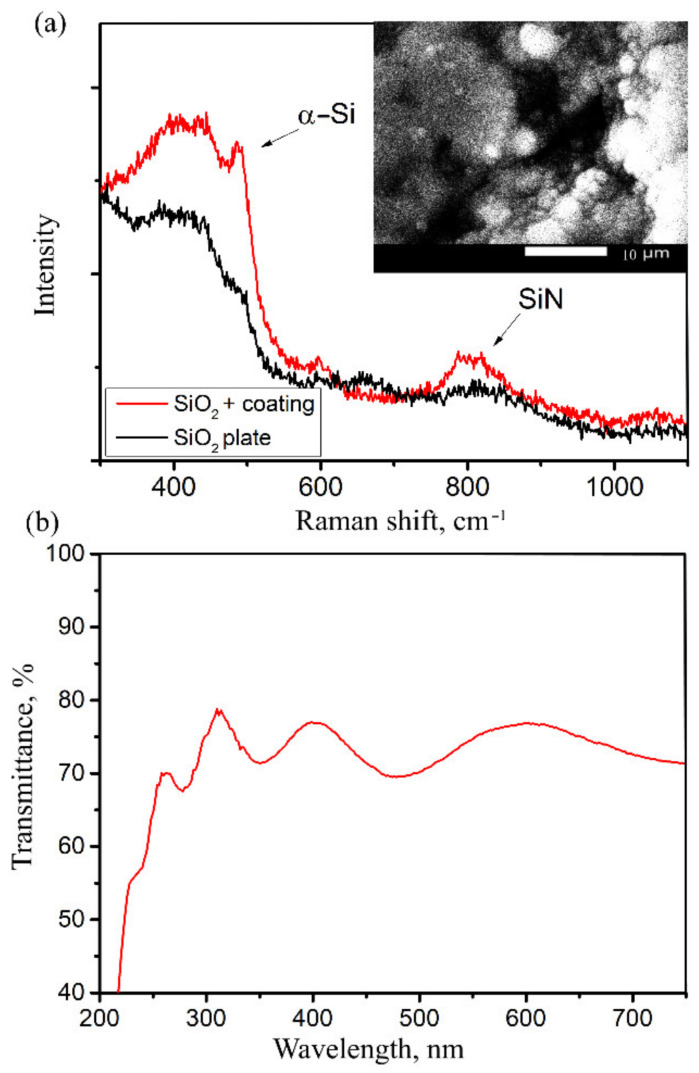
Characterization of silica plates with deposited silicon oxynitride: (**a**) Raman spectrum with SEM−image of the surface (inset), (**b**) transmission spectrum.

Figure 3 illustrates the results of the study of coated silica plates. 

The Raman spectrum demonstrates a typical shape intrinsic to amorphous silicon oxynitride film with a distinct wide Si-N line in the region of 800 cm^−1^, which is consistent with data from the literature [38,39,40]. The same holds for the transmission spectrum of a film indicating to an absorption edge at a wavelength of 245 nm, which corresponds to a band gap of about 5 eV. The band gap can differ significantly, depending on the amount of nitrogen in the resulting oxynitride film [41]. The transmission spectrum was obtained by transillumination of a thin-film coating on the surface of a quartz plate.

The results of the quantitative measurement of oxygen in combination with nitrogen and silicon via electron dispersive microanalysis (EDX) is shown in Table 1.

From the data obtained, it turns out that three parts of silicon account for 4.5 parts of nitrogen and one part of oxygen. Since silicon tetrachloride serves as a precursor, an insignificant amount of chlorine is present in the resulting film.

According to ellipsometric measurements, the real part of the dielectric constant of the resulting coating decreases from 2.2 at a wavelength of 300 nm to 2.02 at 1000 nm. The presence of oxygen decreases the refractive index of the nitride film and the greater the oxygen consumption, the smaller the refractive index. According to the data presented in the literature [42], such a magnitude of the refractive index is typical for oxynitride films with a nitrogen content of more than 80%. The imaginary part of the refractive index in the tested samples 0.2 was from 300 nm to 1000 nm. The obtained set of experimental data confirms that the synthesized material is silicon oxynitride. The thicknesses of the obtained coatings were determined via ellipsometry as well using theoretical models [43]. The obtained film thicknesses were used to determine the deposition rate of the coating for each separate deposition regime.

**Figure 4:**
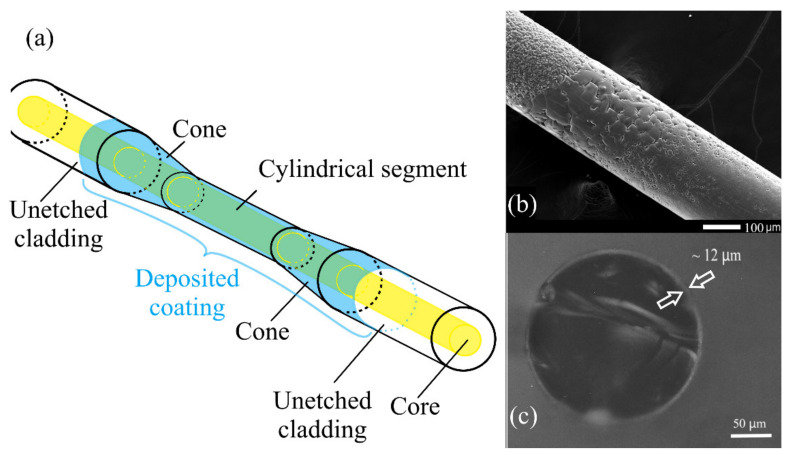
The sensitive part of the fiber: (**a**)—diagram of the thinned part of the fiber, (**b**)—SEM image of the surface, (**c**)—a picture of the end of a taper with a film, magnified in an optical microscope.

Figure 4 shows a schematic diagram of the thinned part of an optical fiber, along with photographs of the surface of the cylindrical part on the applied coating with a thickness of about 12 μm.

Figure 4b illustrates the side surface of a fiber taper coated with a silicon oxynitride film about 12 microns thick. With such a film thickness, an island type of growth of the silicon oxynitride coating can be traced. Figure 4c shows the end face of a chipped taper with a film about 12 microns thick. Such a large thickness of the coating made it possible to visually confirm the high uniformity in thickness over the entire cylindrical surface of the taper. The SEM image captures both the cylindrical region and the beginning of the conical region. Due to the poor quality of the surface on the cone caused by etching, the coating is not uniform and is more island-like than on a cylindrical smooth surface. The main effect on the quality of the sensor will be exerted only by the cylindrical surface of the taper, where the main mode interaction occurs.

**Figure 5:**
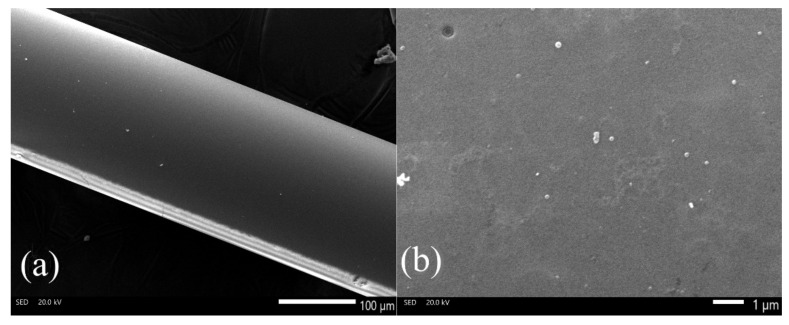
Characterization of fiber samples: (**a**)—SEM-image of a cylindrical surface of a taper with a film, (**b**)—photograph with high magnification.

However, fiber sensors require thinner coatings, in the order of 100–300 nm. Figure 5 shows photographs of the cylindrical part of the sample used as a sensor. The length of the taper for sample #1 was 10 mm, the diameter of the cylindrical part was 205 microns, and the thickness of the coating film was about 220 nm.

One can see that the film is deposited uniformly, and the film is uniform over the entire cylindrical surface, which is important for the sensor. A more uniform and smoother surface allows for better sensor performance, such as sensitivity, resonance shape, and separation of high-order resonances.

### 3.3. Sensor Characterization 

**Figure 6:**
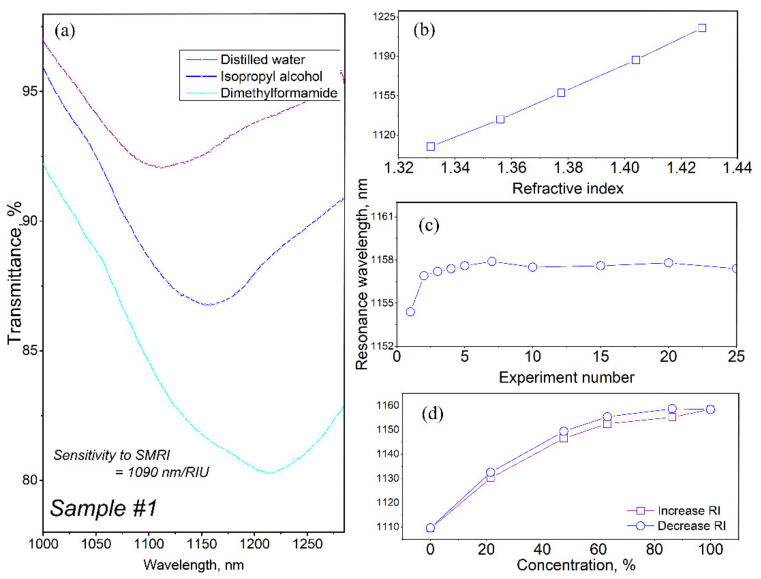
Characteristics of sample #1: (**a**)—transmission spectrum depending on the medium covering the sensor, (**b**)—dependence of the spectral position of the LMR on the refractive index of the environment, (**c**)—resonance position during repeated filling with isopropyl alcohol, (**d**)—shift of the resonance position in aqueous solutions of isopropyl alcohol.

Before evaluating the sensitivity of the obtained sensors, the repeatability of the results was estimated. To do that, the film-covered taper was immersed several times in distilled water and the spectral position of the LMR was measured. A dip in the transmission spectrum of a coated thin section of fiber appears when there is resonance between the HE_1,1_ mode of the optical fiber and the (TE or TM) modes of the supported coating. The spectral position of the resonance depends on the optical characteristics of the fiber, the coating, and the environment, and shifts when any of them changes. With an increase in the refractive index of the external environment (analyte), the resonance shifts to a longer wavelength region. This shift is used to determine the change in the refractive index. As soon as the LMR position after a tenfold immersion remained unchanged, we proceeded to sensitivity testing using solutions of isopropyl alcohol and dimethylformamide. Figure 6 shows the transmission spectra of the sensor in various liquids, as well as the dependence of the LMR position on the refractive index of the environment. The refractive indices of the tested liquids are taken from [44] and correspond to a wavelength of 589 nm.

This thickness corresponds to the first order of LMR resonance. The resulting resonances turned out to be so wide primarily due to the use of a multimode fiber in the experiments, as well as the large length of the thinned part [45]. As one can see, the dependence of the spectral position of the LMR on the refractive index in the studied range of values is almost linear (Figure 6b). To fix the intermediate positions of the resonance, a 30% aqueous solution of isopropyl alcohol (RI = 1.356) and a 30% aqueous solution of dimethylformamide (RI = 1.404) [46] were used. This makes it possible, knowing the position of the edge points, to determine the composition of a certain solution by the refractive index. Repeated pouring of the sample with isopropyl alcohol showed that the position of the resonance shifted slightly during the first few measurements and then stabilized (Figure 6c). Within 25 measurements, the displacement of the resonance position does not exceed 1 nm. This value corresponds to the resolution of the used spectrometer. The resolution of the spectrometer also affects the detection limit. The optical scheme with a sensor used by us allows the change in the refractive index to be fixed by 0.0001. Figure 6d shows the position of the resonance during sequential testing of aqueous solutions of isopropyl alcohol with an increase and subsequent decrease in the concentration of alcohol in the solution. The central points are shifted by a distance of about 5 nm. Since the positions of the resonance in water coincide, the shift is due to a small amount of alcohol in the voids of the film. The sensitivity of the resulting sensor to a change in the refractive index of the environment is 1090 nm/RIU, which significantly exceeds the value of 690 nm/RIU presented in the literature for a sensor on a silicon nitride film [32]. We were unable to find information on similar sensors based on silicon oxynitride coatings. 

Table 2 shows a comparison of the sensitivity of the resulting sensor with others presented in the literature.

In the presented wide range of measured refractive indices, the sensor we obtained is not the most sensitive of those presented in the literature. However, when compared with sensors based on silicon and its nitrides, the result is outstanding. Moreover, the sensitivity is approximately equal to the values obtained on oxide films. However, since oxide films, including titanium oxide or ITO, can only be used for a limited range of media, the material we obtained also has a role. The sensitivity of a resonance to a change in the refractive index depends on many conditions, such as the refractive index of the coating, its roughness and chromatic dispersion. Also important is the wavelength range in which measurements are made, and the width of the range of the measured refractive index. The longer the wavelength and the smaller the range, the higher the sensitivity will be. The combination of the coating material and the analyzed analyte also influences, for example, that oxides are better sorbents than other chalcogenides, therefore they have a higher sensitivity.

**Figure 7:**
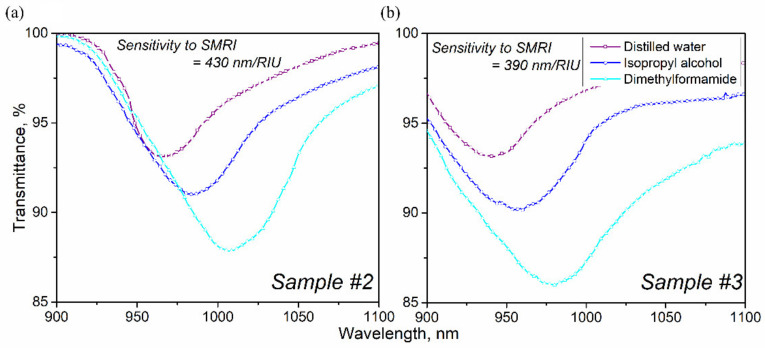
Characteristics of the resonances of the samples: (**a**) #2, (**b**) #3.

The uniformity of the obtained coating, as well as dimensions of the silica reactor, make it possible to synthesize several sensors simultaneously within the framework of a single deposition process. To test this possibility, we redesigned the quartz holder so that two samples of fiber tapers (#2 and #3) could be placed in it at some distance from each other. Sample #2 had a diameter of 205 microns and a length of 4.6 mm, and sample #3 had the same diameter but a length of 5 mm. The target thickness of the film coating was set at the level of 250 nm, as for sample #1. Spectral characteristics of LMR for the samples are shown in Figure 7.

Preliminary testing of both sensors was carried out using the same technique as for sample #1. Subsequent investigation showed that the coating film was slightly thinner than planned, that is, less than 200 nm, but the difference in the initial spectral position of the resonance in water was less than 5%. Using the calculation method [50] described by us earlier, we carried out a theoretical assessment of resonances in samples #2 and #3. Considering the coating parameters obtained from ellipsometric measurements, we found that in distilled water, the difference in film thickness between samples #2 and #3 is about 7 nm. This value lies in the range of non-uniformity of the coating thickness in the deposition zone of the SPCVD method used by us. The sensitivity differed by 15%, which is already less acceptable, but remains quite high among LMR sensors based on nitride [32] and oxynitride film. The sensitivities of samples #2 and #3 amounted to 390 nm/RIU, 430 nm/RIU, respectively. The widths of the resonances differ due to the difference in the length of the tapers, and the shape of the spectra due to the slight difference in the surface morphology. As mentioned earlier, the length of the thinned section of the fiber affects the width and depth of the resonance. Greater length leads to an increase in the width of the dip [45]. 

## 4. Conclusions

A fiber-optic sensor of the refractive index of liquids based on the coating of a double adiabatic fiber taper with a thin film of silicon oxynitride is presented. For the first time, an oxynitride film was applied to a cylindrical surface of a chemically thinned section of a multimode fiber using SPCVD technology. Registration of optical transmission spectra when the sensor is immersed in a liquid with various refractive indices showed the presence of LMR in the telecommunication wavelength range. The resulting sensor demonstrates sensitivity to changes in the refractive index of the external medium at the level of 1090 ± 25 nm/RIU in the range of refractive indices from 1.33 to 1.43. A technology has been developed for the simultaneous fabrication of several sensors with similar characteristics within the framework of a single process for the synthesis of an oxynitride film by means of SPCVD. The difference in the spectral position and depth of the LMR for such twin samples in distilled water does not exceed 5%. The demonstrated technique for synthesizing samples makes it possible to reduce the time required to create several identical sensors and reduce the consumption of precursors. The resulting material has good sensitivity, in comparison with similar ones on oxide films, and has promising properties for use in medicine and biology.

## Figures and Tables

**Table 1:** Quantitative data on the elements content in the synthesized films.

No. Sample	Atomic Percent			
	N	O	Si	Cl
1	53.64	11.49	34.68	0.2
2	53.67	11.39	34.73	0.2
3	53.82	11.41	34.56	0.2
4	53.45	11.4	34.95	0.2

**Table 2:** Sensitivity of different types of sensors, depending on the composition of the coating.

Material	Fiber Geometry	Deposition Method	RI Range	Sensitivity, nm/RIU	Reference
Al_2_O_3_	Temperature adiabatic SMF	ALD	1.33–1.35	6008	[3]
ITO	Side polishing SMF	DC sputtering	1.32–1.38	6009	[12]
In_2_O_3_	Removed cladding MMF	Dip-coating process	1.321–1.372	4920	[14]
ZnO	Side polishing SMF	PLD	1.33–1.458	1700	[47]
TiO_2_/PSS	Coreless MMF	LbL-assembly process	1.32–1.46	955	[48]
Si	Side polishing SMF	PLD	1.33–1.458	270	[49]
SiN_x_	Removed cladding MMF	RF PECVD	1.333–1.4534	690	[32]
Si_3_N_4−x_O_x_	Chemically Etched MMF	SPCVD	1.333–1.43	1090	This work

## Data Availability

Data sharing not applicable.
